# Properties of silicon dioxide layers with embedded metal nanocrystals produced by oxidation of Si:Me mixture

**DOI:** 10.1186/1556-276X-6-148

**Published:** 2011-02-16

**Authors:** Andrei Novikau, Peter Gaiduk, Ksenia Maksimova, Andrei Zenkevich

**Affiliations:** 1 Belarusian State University, 4 prosp. Nezavisimosti, 220030, Minsk, Belarus; 2 NRNU "Moscow Engineering Physics Institute", 31 Kashirskoe shausse, 115409, Moscow, Russian Federation

## Abstract

A two-dimensional layers of metal (Me) nanocrystals embedded in SiO_2 _were produced by pulsed laser deposition of uniformly mixed Si:Me film followed by its furnace oxidation and rapid thermal annealing. The kinetics of the film oxidation and the structural properties of the prepared samples were investigated by Rutherford backscattering spectrometry, and transmission electron microscopy, respectively. The electrical properties of the selected SiO_2_:Me nanocomposite films were evaluated by measuring *C*-*V *and *I*-*V *characteristics on a metal-oxide-semiconductor stack. It is found that Me segregation induced by Si:Me mixture oxidation results in the formation of a high density of Me and silicide nanocrystals in thin film SiO_2 _matrix. Strong evidence of oxidation temperature as well as impurity type effect on the charge storage in crystalline Me-nanodot layer is demonstrated by the hysteresis behavior of the high-frequency *C*-*V *curves.

## Introduction

During the last decade, much attention has been focused on the investigation of semiconductor and metallic nanocrystals (NCs) or nanoclusters embedded in dielectric matrices. The interest is motivated by possible applications of such nanocomposite structures. Particularly, semiconductor or metal NCs embedded in SiO_2 _dielectric layer of a metal-oxide-semiconductor field-effect transistor may replace SiN*_x _*floating gate in conventional Flash memory devices, allowing for thinner injection oxides, and subsequently, smaller operating voltages, longer retention time, and faster write/erase speeds [1-3]. The performance of such memory structure strongly depends on the characteristics of the NCs arrays, such as their size, shape, spatial distribution, electronic band alignment.

Several approaches have been recently tested for the formation of NCs in dielectric layers. Among those, self-assembling of NCs in dielectric layers fabricated by the low-energy ion implantation and different deposition techniques has been studied by several groups [4-7]. A strong memory effect in MOS devices using oxides with Si or Ge NCs was reported in [4,6]. However, the implantation of Ge at the silicon-tunnel oxide interface creates trap sites and results in the degradation of the device performance [4]. The growth technique using MBE deposition of 0.7-1 nm thick Ge layer followed by rapid thermal processing was implemented in [8,9]. An alternative method for Ge NCs production [10] consists of the following steps: low pressure chemical vapor deposition of thin Si-Ge layer, thermal wet or dry oxidation, and thermal treatment in an inert ambient (reduction). Recently, a method to form an ultrathin nanocomposite SiO_2_:NC-Me layers at room temperature by combining the deposition of Si:Me mixed layer on the pre-oxidized Si substrate and its further oxidation in the glow discharge oxygen plasma was proposed [11].

In this article, a similar approach was used to produce thin SiO_2 _layers with an embedded layer of metal NCs. Au and Pt were chosen as metal components in Si:Me mixtures since both metals are believed to catalyze Si oxidation thus reducing the processing temperature, while neither Au nor Pt form stable oxides. Both Pt and Au embedded as NCs in dielectric matrix are attractive materials in plasmonics [12]. In addition, both metals have much higher electron work functions compared to semiconductors, particularly, Ge, and it is interesting to investigate the effect of the NC work function on the electrical properties of the MOS stack with embedded NCs. As the first step, a thin Si:Me layer with the precisely pre-defined composition was grown by pulsed laser deposition (PLD) technique. The oxidation of Si:Me mixture was expected to result in the segregation of the noble metal in NCs distributed in the SiO_2 _matrix. By means of analyzing the Si(O*_x_*):Me elemental depth distributions as a function of the annealing temperature and/or time, we attempted to investigate the kinetics of the composite structure formation. This information was supplemented by microstructural transmission electron microscopy (TEM) analysis and further--by electrical measurements on metal/SiO_2_:Me-NC/Si capacitors.

## Experimental

**Figure 1:**
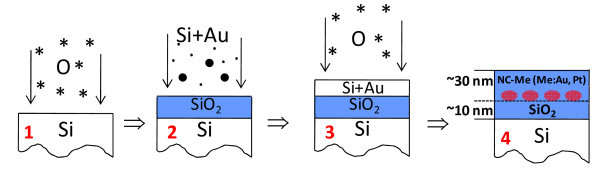
**The proposed procedure of the MOS stack formation including SiO_2 _layers with the embedded metal NCs**.

*N*-type Si(001) wafers were used as substrates. The uniform SiO_2 _layer 6 nm in thickness (tunnel oxide) was first grown in a dry oxygen ambiance. An amorphous Si:Me (Me = Au, Pt) layer 20 nm in thickness was then deposited by PLD at room temperature. The computerized ultra-high vacuum (base pressure *P *= 10^-6 ^Pa) home-made PLD setup employing YAG:Nd laser (λ = 1,064 μm) and operating in the Q-switched regime (τ = 15 ns) at the variable output energies *E *= 50-200 mJ and the repetition rates ν = 5-50 Hz was employed to ablate from the elemental Si and Me (Me = Au, Pt) targets. The pre-calculated composition of the Si:Me mixture necessary to form the desired nanocomposite structure was provided by choosing the exact ratio of Si vs. Me deposition pulses in a deposition cycle during the Si:Me layer growth. The sandwiched Si:Me/SiO_2_/Si samples were further thermally oxidized in dry oxygen ambient. To exclude the coalescence of the segregating metal NCs, the thermal budget should be minimized. Therefore, to determine the minimal temperatures to oxidize Si:Me mixtures at our conditions, the preliminary experiments were performed. It is worth noting that the presence of a noble metal in Si:Me mixture is found to significantly reduce the oxidation temperatures as compared to pure Si. Thus, the chosen oxidation conditions were *T *= 640-725°C for 60-540 min. Finally, the thermally oxidized structures were subjected to rapid thermal annealing in dry nitrogen ambient at *T *= 900°C for 30 s. The sequential processing steps are shown in Figure 1. A reference SiO_2_/Si sample with no metal NCs was prepared for comparison.

The composition of and the metal depth distribution in the samples were measured using Rutherford backscattering spectrometry (RBS) with a He^+ ^beam at *E *= 1.5 MeV. The spectra were taken simultaneously at two different scattering angles, θ = 10° and θ = 75°, with the former geometry being used to calculate the integral metal concentration in Si:Me, while the latter one to observe possible changes in the metal distribution upon oxidation. The experimental spectra were analyzed using the RUMP software [13]. The structural quality and the phase composition were analyzed using the TEM in both plain-view and cross-sectional geometries using a Philips CM20 instrument operating at *U *= 200 kV. MOS capacitors with In electrodes were fabricated, and the high-frequency *C*-*V *measurements were carried out using a serial HP4156B instrument.

## Results and discussion

**Figure 2:**
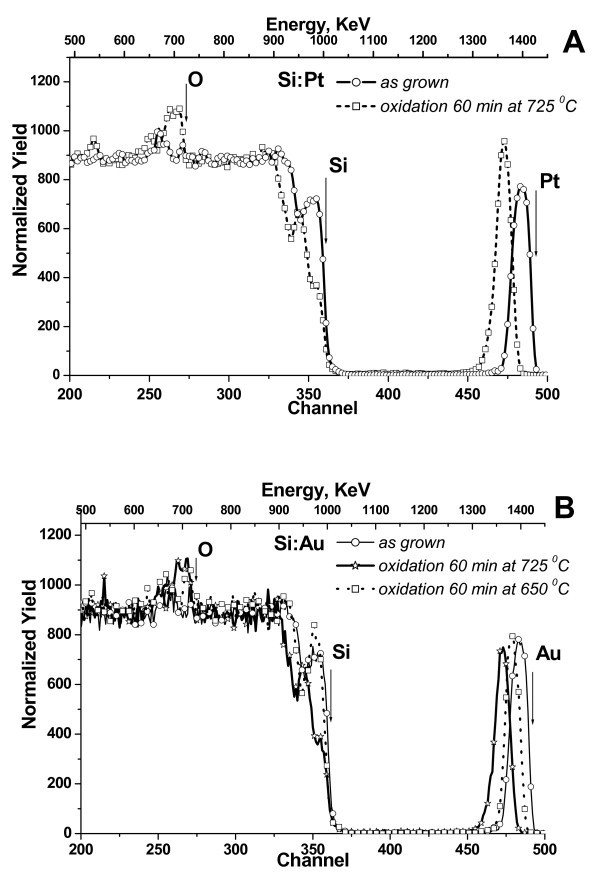
**RBS spectra from as grown and thermally oxidized Si:Me/SiO_2_/Si samples**: **(a) **RBS spectra (*E *= 1.5 MeV, θ = 75°) from Si:Pt/SiO_2_/Si samples thermally oxidized at *T *= 725°C for 60 min in O_2 _followed by thermal annealing in N_2 _at *T *= 900°C for 30 s. as compared with as-grown structure; **(b) **Au peak in RBS spectra evidences strong Au segregation during Si oxidation process at different temperatures.

The typical RBS spectra from the as-grown and thermally treated Si:Me/SiO_2_/Si samples are presented in Figure 2. The RBS spectra show that the thickness of as-deposited Si:Au layers is about 20 nm. The metal concentration in the deposited layers is in the range 2.5-4.5%. The shift of both Au and Pt peaks to the lower energies upon thermal oxidation evidencing the pile up of metal atoms at the SiO_2_/Si interface is clearly observed in RBS spectra. The observed evolution of Pt and Au concentration profiles indicates the complete rejection of Me atoms from the oxide during thermal oxidation of *a*-Si:Me layer. The detailed analysis of RBS data (Figure 2) reveals that Au and Pt segregation depends on the oxidation conditions. In particular, neither evaporation nor diffusion of Au or Pt in SiO_2 _layer takes place during thermal oxidation in dry O_2_. On the contrary, oxidation at higher temperatures results in a strong loss (about 30%) of Me from the SiO_2 _layer, apparently due to evaporation and partial diffusion into the Si substrate.

**Figure 3:**
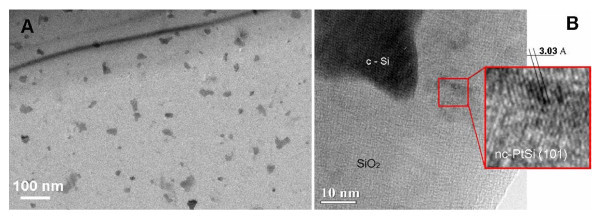
**Transmission electron microscopy analysis from a Si:Pt sample, oxidized at T = 640°C for 5 h in dry O2: bright-field plain-view (a) and high resolution (b) TEM images**. Crystalline PtSi NCs exhibit a dark contrast on the gray background of the SiO_2 _layer.

The results of the plain-view TEM investigations (published elsewhere [14]) correlate well with the RBS data. Figure 3a clearly shows the well-separated clusters embedded in the SiO_2 _layer formed after thermal treatment. The average size and the areal density of the observed NCs were estimated to be from 10 to 20 nm and 2 × 10^10 ^cm^-2^, respectively. To elucidate the structural properties of metal NCs, the HRTEM analysis was performed. The results for SiO_2_:NC-Pt are shown in Figure 3b. The bright-field TEM micrograph of the Si:Pt-alloyed sample oxidized at *T *= 640°C for 5 h reveals dark-gray clusters scattered on a light gray SiO_2 _background. Careful examination of the clusters structure performed using the direct resolution of crystallographic planes and selected area electron diffraction patterns analysis (not shown) evidences the formation of platinum monosilicide (PtSi) crystalline phase in NCs. In addition, unoxidized silicon islands were also identified. Similar results were also obtained for Si:Au samples although no evidence of Au silicide formation was found (not shown). A previous study [11] describing detailed *in situ *investigation by X-ray photoelectron spectroscopy of the Au chemical state evolution during the oxidation of the similarly produced Si:Au mixture indicated the formation of a metastable Au silicide during the room temperature deposition and its further decomposition to metallic Au upon oxidation.

The self-assembling phenomenon of the formation of metal and silicide NCs in SiO_2 _can be explained using two mechanisms. A solubility of impurities in SiO_2 _is quite low, and therefore the structures obtained after metal segregation and piling up between two SiO_2 _layers (tunnel oxide and SiO_2 _capping layer) were transformed into the supersaturated solution. It is well known that under the thermal treatment the decomposition of supersaturated solution takes place eventually resulting in the phase separation and the formation of the metal NCs in a dielectric (oxide) matrix. On the next stage, the Ostwald ripening of the formed NCs occurs. This implies the diffusion of metal atoms from the valley regions of the islands toward their respective centers forming spherical nanocrystals to achieve greater volume-to-surface ratio. In our model, the initial NCs are formed during the oxidation of the Si:Me layer. After the oxidation is completed, the sample is still kept at elevated temperature facilitating the coalescence of Me NCs.

**Figure 4:**
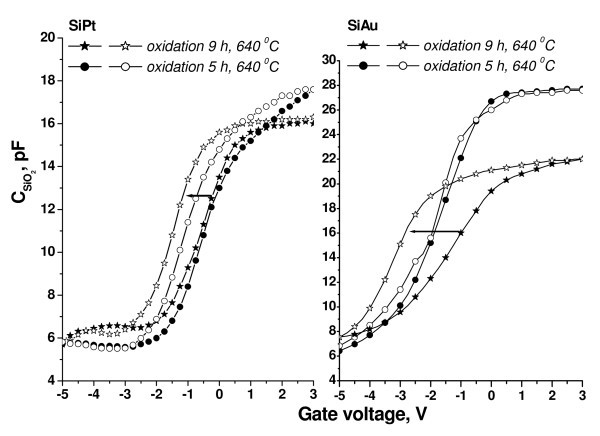
**High-frequency *C*-*V *curves measured from Si:Au and Si:Pt samples, oxidized at *T *= 640°C for 5 and 9 h in dry O_2_, respectively**. A gate voltage sweep from inversion to accumulation and from accumulation to inversion is shown on the figure by arrows.

The effect of the oxidation temperature as well as the type of the embedded Me on the efficiency of the charge storage was studied by the high-frequency *C*-*V *measurements. The hysteresis in *C*-*V *curves was found different for the structures containing Au and PtSi NCs (Figure 4). The maximal value of the flat-band voltage shift *U *= 1.8 V for the *V*_g _sweep -5/+3 V was obtained for SiO2:NC-Au based structures prepared by dry oxidation. On the contrary, in the case of SiO_2_:NC-PtSi, the maximal flat-band voltage shift was *U *= 1.2 V. By increasing *V*_g _sweep up to 5 V, a gradual increase of the flat-band voltage shift was achieved. Since high positive gate voltages shift *C*-*V *curves in the direction of the stored negative charges, it is concluded that the charge trapping occurs through the electron injection from the substrate into the oxide. No flat-band voltage shift was observed for the reference sample prepared with pure SiO_2_, oxidized at *T *= 850°C for 60 min in O_2 _ambient. It is therefore concluded that the effect of charge storage is related to the NCs.

**Figure 5:**
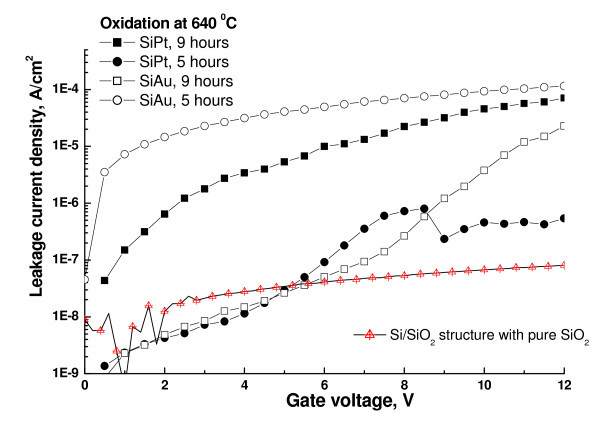
**Leakage current vs. gate voltage characteristics obtained from the oxidized Si:Au and Si:Pt samples at *T *= 640°C**. The *I*-*V *curve from the reference sample of pure SiO_2 _is shown for comparison.

One of the major reasons for the loss of charge in the floating gate structures is the leakage current. The measured *I*-*V *curves (Figure 5) from Si:Au and Si:Pt samples oxidized in dry ambient reveal that the leakage current density can be reduced down to 10^-8 ^A/cm^2^. The low leakage currents achieved are explained by the high quality of both tunneling and capping oxide formed by dry thermal process compared with the deposited oxides used in the alternative methods of MOS capacitor formation [15]. It is found that the oxidation temperature has also a strong effect on the leakage current, and therefore the oxidation conditions should be optimized for each type of embedded metal NCs.

## Conclusion

In this study, the authors have demonstrated the growth of thin SiO_2 _layers with embedded metal and metal silicide NCs by the combination of Si:Me mixture by PLD at room temperature and its thermal oxidation. By means of this fabrication technique, it is possible to produce a sheet of crystalline metal nanocrystals at any desirable depth in the oxide. The metal segregation process during thermal oxidation results in the formation of a high areal density of crystalline Au and PtSi dots 10-20 nm in diameter which are distributed in the silicon dioxide at a distance of 5-6 nm from the crystalline Si substrate. The charge storage effect is evident from *C*-*V *characteristics on MOS capacitors, and the results indicate the injection of the electrons from the substrate. The flat-band voltage shift of about 1.2-1.8 V for *V*_g _sweeps of -5/+3 V is achieved. It is shown that the leakage current density depends mostly upon the oxidation conditions, and for both types of metal NCs (Au and PtSi), it was measured to be around 10^-8 ^A/cm^2^. The reproducibility and the precision of the proposed fabrication technique (PLD and thermal treatment) to produce a 2 D array of well-separated nanocrystals in a SiO_2 _layer suggest that this method can be applied for the fabrication of functional MOS structures.

## Abbreviations

NCs: nanocrystals; PLD: pulsed laser deposition; RBS: Rutherford backscattering spectrometry; TEM: transmission electron microscopy; MOS: metal-oxide-semiconductor.

## Competing interests

The authors declare that they have no competing interests.

## Authors' contributions

AN participated in the RBS analysis and carried out the electrical characterization, participated in the design of the study and drafted the manuscript. KM carried out the pulsed laser deposition and experimental data analysis. PG conceived of the study, and participated in its design and coordination. AZ participated in the design of the study, coordinated TEM analysis and significantly contributed to the writing of manuscript. All authors read and approved the final manuscript.
